# Influenza Mortality in the United States, 2009 Pandemic: Burden, Timing and Age Distribution

**DOI:** 10.1371/journal.pone.0064198

**Published:** 2013-05-22

**Authors:** Ann M. Nguyen, Andrew Noymer

**Affiliations:** 1 Palomar Health, Escondido, California, United States of America; 2 Department of Population Health and Disease Prevention, University of California Irvine, Irvine, California, United States of America; University of Hong Kong, Hong Kong

## Abstract

**Background:**

In April 2009, the most recent pandemic of influenza A began. We present the first estimates of pandemic mortality based on the newly-released final data on deaths in 2009 and 2010 in the United States.

**Methods:**

We obtained data on influenza and pneumonia deaths from the National Center for Health Statistics (NCHS). Age- and sex-specific death rates, and age-standardized death rates, were calculated. Using negative binomial Serfling-type methods, excess mortality was calculated separately by sex and age groups.

**Results:**

In many age groups, observed pneumonia and influenza cause-specific mortality rates in October and November 2009 broke month-specific records since 1959 when the current series of detailed US mortality data began. Compared to the typical pattern of seasonal flu deaths, the 2009 pandemic age-specific mortality, as well as influenza-attributable (excess) mortality, skewed much younger. We estimate 2,634 excess pneumonia and influenza deaths in 2009–10; the excess death rate in 2009 was 0.79 per 100,000.

**Conclusions:**

Pandemic influenza mortality skews younger than seasonal influenza. This can be explained by a protective effect due to antigenic cycling. When older cohorts have been previously exposed to a similar antigen, immune memory results in lower death rates at older ages. Age-targeted vaccination of younger people should be considered in future pandemics.

## Introduction

In April 2009, a novel strain of influenza A/H1N1 emerged in Mexico, rapidly spreading to the United States [1] and then worldwide. On 11 June 2009, the World Health Organization declared a pandemic [2], which eventually caused an estimated 284,500 deaths worldwide [3]. The pandemic was declared over on 10 August 2010 [4]. Approximately 20% of the US population contracted influenza during the pandemic [5], [6]. In 2009, there were 53,692 pneumonia and influenza deaths, making this combination the eighth leading cause of death [7]; in 2010 there were 50,003 pneumonia and influenza deaths (ninth leading cause) [8]. Overall, the pandemic case fatality rate was low, and attack rates were higher among children and young adults [9]. The sparing of adults 65 and older is thought to be due to immunity from previous exposure to antigenically-similar H1N1 strains [10]–[15].

In this article, we analyze final mortality data for the United States which were released in August 2012. This is the first analysis of the complete mortality record of influenza for 2009 and 2010. Our age- and sex-specific analysis incorporates data from 1959–2010, and pneumonia and influenza deaths were analyzed together [16]. Age-specific mortality burden estimates can aid pandemic planning.

## Materials and Methods

All the data used in this study are fully in the public domain. We obtained data on number of deaths, by cause, from the mortality detail files of the National Center for Health Statistics (NCHS) [17]. Deaths were stratified by age, sex, month, and underlying cause. We extracted data on influenza and pneumonia deaths from January 1959 to December 2010. As shown in table 1, this period spans four revisions of the International Classification of Diseases (ICD 7–10). To ensure comparability, all data were converted to ICD-10 using the published crossover tables [18]–[20]. Prior to 1959, no detailed mortality data (i.e., simultaneously disaggregated by age, sex, month, and cause) are available. Final mortality data for 2009 and 2010 were released in 2012.

**Table 1:** ICD codes for Pneumonia & Influenza.

	Causes used as
Years	Pneumonia & Influenza
1959–1967 (ICD 7)	480–483, 490–493
1968–1978 (ICD 8)	470–474, 480–486
1979–1998 (ICD 9)	480–487
1999–2010 (ICD 10)	J10–J18

Codes from the four revisions of the ICD (International Classification of Diseases) merged to the combined cause “pneumonia and influenza.”

Rates (per 100,000) were calculated using these death counts in the numerator, and exposure data (person-years at risk) from the Human Mortality Database [21] in the denominator. The age-specific exposure data were interpolated to monthly units to match the death counts. Variable days per month, including leap years, were used in the monthly exposure interpolation.

Excess mortality was calculated using modified Serfling-type methods [22], [23] on the monthly death rates described above, with negative binomial regression in place of ordinary least squares [24]–[26]. Our excess mortality calculation was done separately by age groups, which permits full use of the data, while being collapsible to all-ages in a robust way [27]. We used a Serfling approach similar to that of [28] (except using negative binomial regression), running regressions of the type:
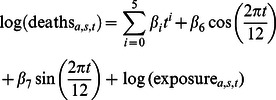
where subscripts 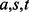 denote age- and sex-specific models, with monthly time (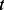) resolution. The model de-trends by a quintic polynomial in time (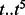), and includes two harmonic (sin, cos) terms; 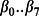 are coefficients to be estimated. In keeping with convention for count models (such as Poisson and negative binomial regression), the log rate is decomposed into the difference of log deaths and log exposure, with the latter being moved to the right hand side of the estimating equation; this is equivalent to regressing log rates [29].

Count regression models produce estimates that are broadly comparable to classic Serfling methods [30], but are a refinement. Negative binomial regression takes into account the fact that deaths are counts, and may be overdispersed. Moreover, negative binomial regression performs well with low counts, so is suited to sex- and age-disaggregated analysis. The models establish a baseline by estimating a waveform from the summer troughs (May–October) of pneumonia and influenza mortality. September and October 2009, however, were excluded from the baseline calculations due to the atypically early circulation of influenza virus during this time period.

Upper and lower bounds were calculated from jacknifed standard errors [29] to create a 95% confidence bound. Excess mortality is the observed deaths minus the baseline prediction. An analogous approach to excess mortality calculation is to use all-cause mortality in lieu of pneumonia and influenza, but we chose to emphasize specificity over sensitivity [31]. Analysis was done using IDL version 8.2 (Exelis Visual Information Solutions, Inc., Boulder CO, USA) and Stata version 10.1 (StataCorp LP, College Station TX, USA).

## Results

**Figure 1:**
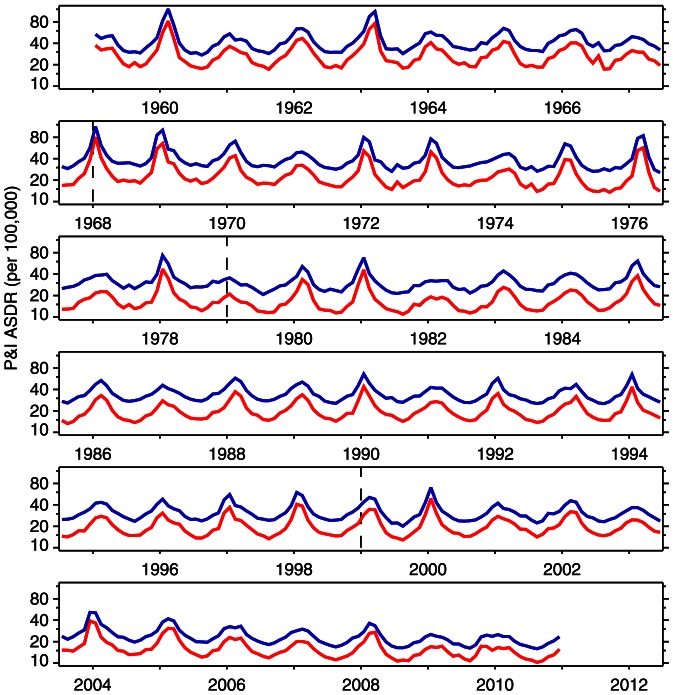
Monthly age-standardized death rate (ASDR) for pneumonia and influenza, United States, 1959–2010. Males blue, females red. Dashed lines denote boundaries of ICD regimes, but data are adjusted for ICD changes.

Figure 1 plots the age-standardized death rate (ASDR) for pneumonia and influenza for the United States from January 1959 to December 2010. This figure presents the most complete monthly record of pneumonia and influenza ASDR in the US. Considering death rates weighted over all ages, recent influenza pandemics are not especially severe. This is seen clearly in figure 1 for 2009, as well as for the H3N2 “Hong Kong” influenza pandemic of 1968–69 [32]. Due to the fall wave of the 2009 H1N1 pandemic, the mortality of the winter 2009–10 influenza season began unusually early [33], creating a plateau-like flu season. Calendar year 2003 is the only other recent year with pneumonia and influenza mortality rising so strongly in the fall, in association with the emergence of the Fujian strain of influenza A/H3N2 [34]. Other remarkable features of figure 1 are the secular decline of the ASDR for pneumonia and influenza, and the consistency with which male death rates exceed those of females.

**Figure 2:**
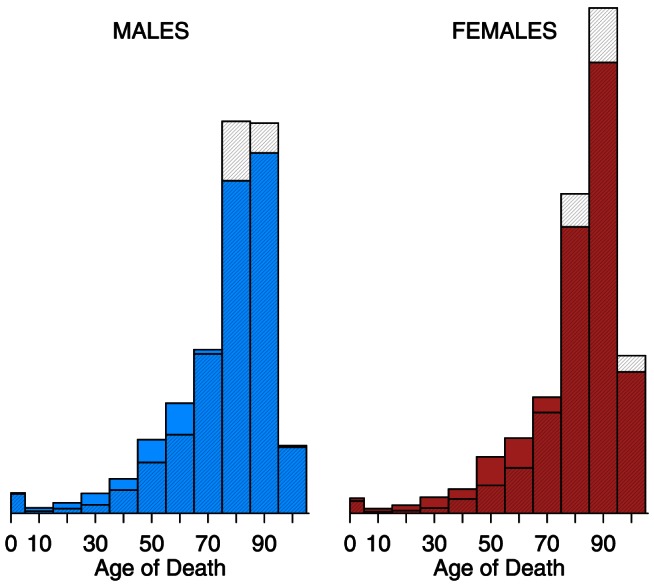
Histograms of pneumonia and influenza (ICD 10 J10–J18) deaths by age, 2009 vs. 1999–2008. Year 2009 is solid; 1999–2008 average is cross-hatched.

**Figure 3:**
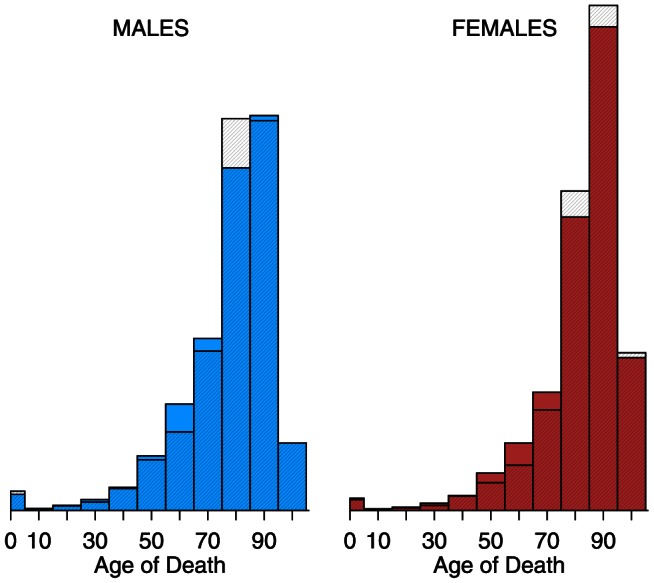
Histograms of pneumonia and influenza (ICD 10 J10–J18) deaths by age, 2010 vs. 1999–2008. Year 2010 is solid; 1999–2008 average is cross-hatched.

Figures 2 and 3 present the distribution of pneumonia and influenza deaths for calendar years 2009 and 2010, separately, by age group (0–4, 5–14, , 85–94, 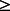
95) and by sex. The solid bars represent the calendar year, and, for comparison, the cross-hatched bars are the average of 1999–2008. In 2009, mortality skewed younger compared to the previous 10-year average: the solid bars exceed the hatched bars for all ages 0–74, for both sexes. In 2010, the distribution of mortality resembled the 10-year average. Both female histograms skew older than those for males. Although females consistently have lower pneumonia and influenza death rates than males, their longer life expectancy [7] means more females at older ages, and therefore more deaths at older ages.

**Figure 4:**
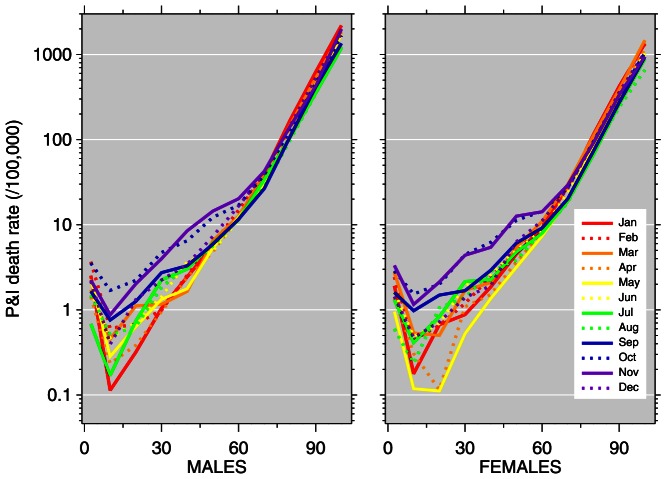
Pneumonia and influenza (P&I) age-mortality profiles by month, 2009. Vertical axis: P&I death rate per 100,000; horizontal axis: age.

**Figure 5:**
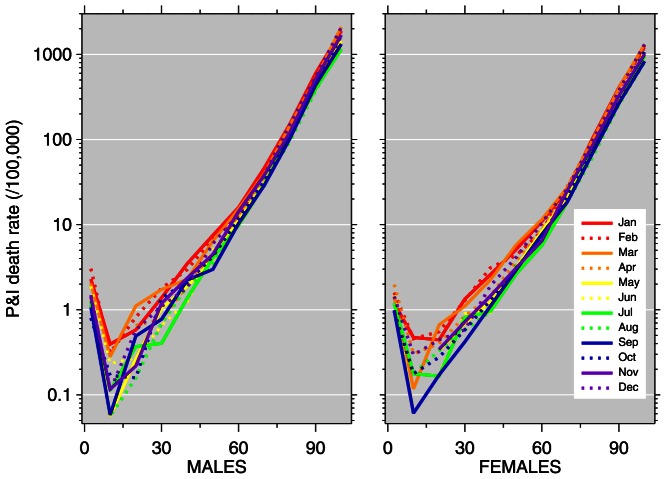
Pneumonia and influenza (P&I) age-mortality profiles by month, 2010. Vertical axis: P&I death rate per 100,000; horizontal axis: age.

Figures 4 and 5 plot pneumonia and influenza death rates versus age for each month of 2009 and 2010, separately by sex. This illustrates combined aspects of figure 1 (unusual timing) and figure 2 (younger age distribution) of the 2009 pandemic. In 2009, unusually high death rates in young age groups were recorded in October through December. In 2010, elevated death rates from the 2009 pandemic continued, with young ages impacted in January through March. The nadir of the 2010 graphs comes in September, with pneumonia and influenza death rates below 1 per million (i.e., 0.1 per 100,000) for the age group 5–14; comparatively, in 2009, death rates in this age group were approximately ten times higher.

As table 2 details, October–November 2009 is one of the most remarkable periods in the history of pneumonia and influenza mortality since 1959, especially for females. These comparisons refer to mortality rates, not death counts. October 2009 recorded the highest pneumonia and influenza death rate for females in the age span 25–54, out of all Octobers, 1959–2010. This month was less notable for males, although in ages 25–34 they also had record mortality rates. Likewise, November 2009 recorded record-high pneumonia and influenza death rates: for females, in the age span 15–54, and for males, 25–34. December 2009, on the other hand, did not experience any record high mortality rates. Mortality rates for 2010 also did not experience any record highs. Prior to 1959, age
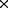
sex
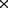
cause-of-death monthly data are unavailable, so the October–November record-high death rates are at least 52-year records.

**Table 2:** October and November record-high P&I mortality rates.

	Males				Females			
Age	Octobers		Novembers		Octobers		Novembers	
Group	Rate	Year	Rate	Year	Rate	Year	Rate	Year
0–4	42.61	1959	54.96	1959	36.99	1960	44.75	1959
5–14	1.90	1965	2.01	1962	2.00	1960	2.39	1962
15–24	2.31	1969	2.77	1959	2.40	1959	2.12	2009
25–34	4.72	2009	3.98	2009	4.47	2009	4.38	2009
35–44	6.74	1964	8.58	1968	6.21	2009	5.42	2009
45–54	15.11	1964	16.60	1968	11.28	2009	12.61	2009
55–64	29.22	1965	34.23	1968	15.00	1962	14.76	1960
65–74	79.75	1961	83.18	1965	36.68	1965	39.19	1959
75–84	263.29	1965	266.30	1965	145.24	1964	164.45	1961
85–94	935.29	1987	947.96	1993	566.80	1987	606.07	1962
≥95	2792.80	1989	3107.13	1987	1789.01	1961	1972.49	1991

Record-high mortality rates (per 100,000) for 11 age groups in Octobers and Novembers, 1959–2010. Out of 44 age
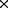
sex
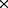
month combinations, 9 record highs occur in 2009, all below age 55.

**Figure 6:**
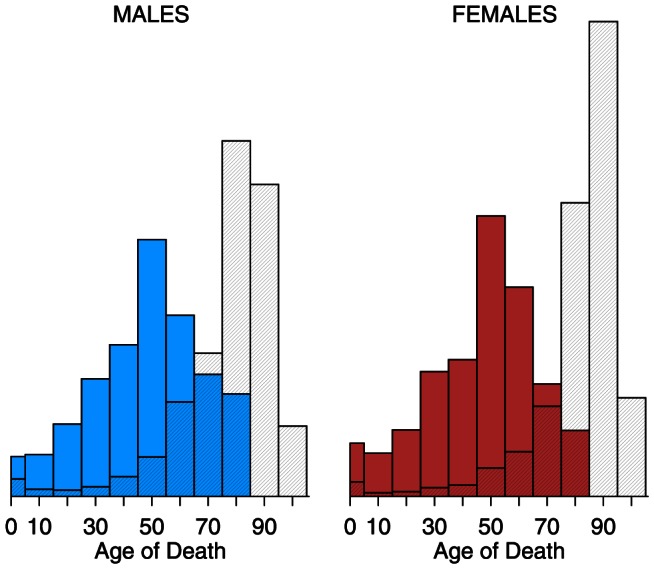
Histograms of pneumonia and influenza excess deaths by age, 2009 vs. 1999–2008. Year 2009 is solid; 1999–2008 average is cross-hatched.

**Figure 7:**
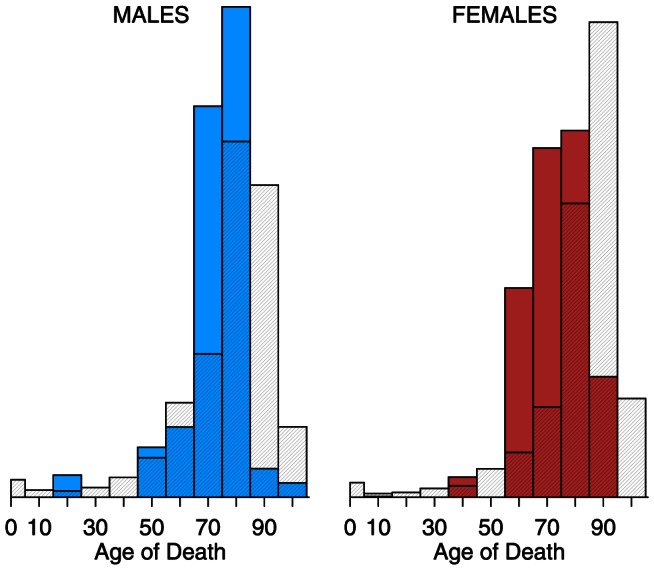
Histograms of pneumonia and influenza excess deaths by age, 2010 vs. **1999–2008.** Year 2010 is solid; 1999–2008 average is cross-hatched.

Figures 6 and 7 display the age distribution of influenza-attributable excess mortality as calculated by the negative binomial Serfling regression model of pneumonia and influenza death rates. The age groups and shading follow that of figures 2–3. Even more so than age-specific mortality (i.e., figure 2), 2009 excess mortality skews young. Thus, the youngness of the distributions in 2009 (solid) versus 1999–2008 (cross-hatched) reflects the pandemic, and is not an artifact of switching the metric from raw mortality (figure 2) to excess mortality (figure 6). In 2009, the solid bars exceeded the cross-hatched bars from ages 0–64 for both sexes. In 2010, the distribution of excess mortality more closely resembled the 10-year average, though still with a slight skew away from the oldest ages.

Table 3 provides excess pneumonia and influenza mortality estimates by age group and sex, with the corresponding 95% confidence bounds, for 2009 and 2010. Negative excess mortality values indicate that the observed pneumonia and influenza deaths in the age group were less than the age group-specific negative binomial Serfling baseline. It can also be an indication that the model is an inadequate estimate of the appropriate baseline; this is an intrinsic limitation of Serfling-type methods. Where the bound includes zero, it may be interpreted as non-statistically-significant excess mortality. In 2009, excess mortality is highest in the age group 45–54 for both males and females. The negative excess mortality in the 85–94 age group for males and 85 and above for females is notable. We estimate 2,438 total excess deaths in 2009. In 2010, excess mortality is highest in the age group 65–74 for males and 55–64 for females. We estimate 196 total excess deaths in 2010; for females, however, the total excess deaths were lower than that expected from the negative binomial baseline.

**Table 3:** Excess mortality estimates.

	Males		Females	
Age Group	Excess Mortality	(lower bound, upper bound)	Excess Mortality	(lower bound, upper bound)
2009				
0–4	30.5	(29.4, 31.6)	34.3	(33.1, 35.4)
5–14	59.2	(56.2, 62.3)	63.5	(60.5, 66.5)
15–24	104.3	(102.6, 106.1)	84.2	(81.5, 86.9)
25–34	171.5	(169.9, 173.1)	164.9	(162.8, 167.1)
35–44	242.8	(241.6, 243.9)	189.8	(188.5, 191.1)
45–54	383.8	(383.1, 384.4)	424.5	(423.7, 425.3)
55–64	283.6	(283.3, 283.9)	291.5	(291.0, 292.0)
65–74	126.2	(125.9, 126.5)	164.4	(164.1, 164.7)
75–84	96.3	(96.0, 96.5)	92.3	(92.0, 92.5)
85–94	−222.0	(−222.3, −221.8)	−185.0	(−185.3, −184.8)
≥95	24.0	(23.6, 24.4)	−186.5	(−186.9, −186.1)
Total	1300.2	(1289.3, 1310.9)	1137.9	(1125, 1150.6)
2010				
0–4	−0.8	(−1.7, 0.0)	−4.6	(−5.5, −3.8)
5–14	−0.1	(−2.2, 1.9)	5.9	(3.7, 8.0)
15–24	23.9	(22.6, 25.2)	6.6	(4.8, 8.4)
25–34	12.0	(10.9, 13.1)	12.7	(11.2, 14.2)
35–44	−0.7	(−1.5, 0.1)	34.5	(33.6, 35.4)
45–54	59.7	(59.3, 60.2)	18.1	(17.5, 18.6)
55–64	59.0	(58.7, 59.3)	63.0	(62.6, 63.4)
65–74	128.7	(128.5, 129.0)	37.2	(36.9, 37.4)
75–84	117.5	(117.3, 117.7)	−4.8	(−5.0, −4.6)
85–94	−116.4	(−116.6, −116.1)	−47.8	(−48.0, −47.5)
≥95	−11.6	(−12.0, −11.2)	−196.2	(−196.6, −195.9)
Total	271.2	(263.3, 279.2)	−75.4	(−84.8, −66.4)

Excess pneumonia and influenza mortality estimates (deaths) and 95% confidence bounds for calendar years 2009 and 2010.

**Figure 8:**
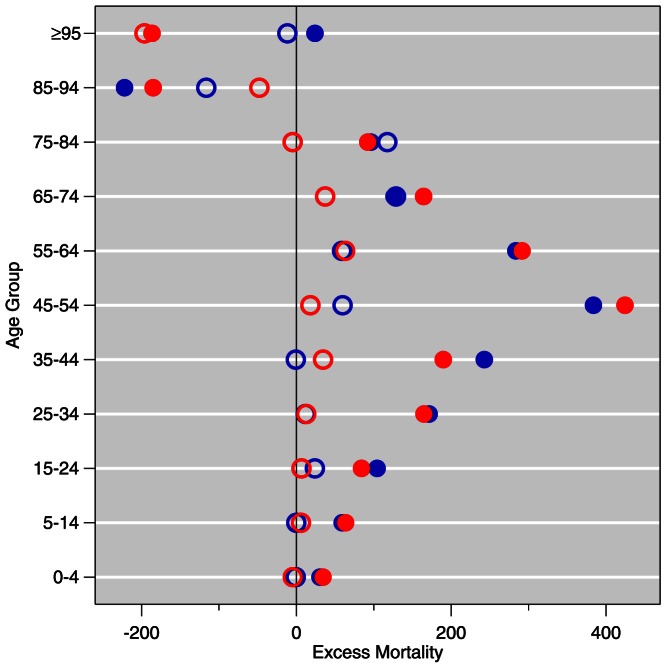
Comparison of 2009 and 2010 pneumonia and influenza excess deaths by age. Males blue, females red. Solid circles 2009, open circles 2010.

The estimates from table 3 are illustrated in figure 8. To address the relative weight of various age groups, the histograms (density estimators) in figures 6–7 show the age distribution of excess mortality [35]. Figure 8, on the other hand, is more suited to quantitative comparison of excess deaths from age to age. The solid circles represent excess mortality estimates for 2009, and the open circles are for 2010. Whiskers were not drawn because the jacknifed negative binomial standard errors resulted in narrow confidence bands (cf. table 3). Excess mortality peaked at age group 45–54 in 2009, whereas in 2010 it peaked lower and older.

## Discussion

The public health concern about influenza pandemics stems, above all else, from the potential to kill millions, as in the 1918–19 pandemic [36]. The mortality of the 2009 pandemic was unusual not for its mortality burden but for its timing and age distribution. In October and November 2009, pneumonia and influenza mortality rates in a number of age groups were the highest for those months since at least 1959. This phenomenon, which occurred exclusively below age 55, was more pronounced for females; the reasons for the sex difference are unclear. Similarly, there is no salient explanation for the sex differences observed in the 2010 excess death distribution by age. Sex differentials in influenza infection [37]–[40] and mortality during pandemics are topics of current interest [41], [42].

Seasonal influenza typically kills with an old-age mortality pattern: the cross-hatched bars (1999–2008 average) of figures 2–3 and 6–7 peak at age 75 and above. There were three influenza pandemics in the twentieth century (1918–19, 1957, 1968–69) [43], all of which skewed young [44]–[49]. The 2009 and 2010 data confirm this pattern. There is a limited sample from which to draw conclusions, but influenza pandemic mortality consistently skews young. The younger pattern is seen in both 2009 age-specific mortality (figure 2, figure 4, and table 2) and excess mortality (figure 6, figure 8, and table 3). Our finding is consistent with earlier work [50], [51]. Excess mortality is calculated relative to a baseline model [22], [23]; thus, excess mortality estimates may be influenced by timing (phase) as well as severity (amplitude). The age-specific mortality data are not model-based, so the qualitative agreement of younger skew in both modes of analysis suggests that the skew is not an artifact.

Overall, 2009 had about 2,438 pneumonia and influenza excess deaths, and 2010 had about 196. Our estimated total excess deaths is 2,634. The bulk of pneumonia and influenza mortality typically occurs above age 60, but when the pandemic strain of influenza H1N1 displaced other circulating strains [52], the accompanying rise in pneumonia and influenza mortality took place in age groups which typically have low mortality rates. The unusual mortality at young ages is a remarkable indication of the specific impact of the pandemic. Our estimates are more conservative than other estimates of mortality burden [5], but this should not be regarded as especially unusual since multiplier models (see also [53]) and modified Serfling-type models represent different approaches. Although the present study focuses on sex- and age-distribution, our estimates collapse to a total burden of 0.79 excess deaths per 100,000 population in 2009. The comparison of excess death estimates produced using different methods is difficult [54], but our estimate is similar to published estimates for other countries, such as France (0.98 per 100,000 [55]), Denmark (2.19 per 100,000 for winter [56]), Hong Kong (2.20 per 100,000 for winter [57]), and Mexico (3.9 per 100,000 for April–December 2009 [58]).

After reemergence in 1977, influenza A/H1N1 spread in children and young adults because of its remarkable antigenic similarity to the pre-1957 H1N1 strains [59], [60]. The protective effect of older cohorts having seen this strain before was clear-cut. In the case of 2009, an analogous phenomenon is likely [10], [11]. This may be an example of original antigenic sin [61]–[66]. Our estimates show negative excess mortality among both sexes above age 85 in both 2009 and 2010, with the exception of males 95 and older in 2009, where excess mortality was positive but low. A likely explanation is a protective effect of prior exposure to H1N1 influenza viruses which were in circulation between 1918 and 1957 [67]–[69].

This study is not without limitations. We use only publicly-available data and therefore the time resolution is monthly. Although we employed negative binomial regression as an update to classical Serfling analysis, the present study shares the limitations of any technique that analyzes mortality data by itself without bringing in viral surveillance co-variates [70] or influenza-like illness (ILI) morbidity. We chose to focus on pneumonia and influenza mortality as a combined cause, emphasizing specificity over sensitivity [31] (e.g., a flu-related death coded under a heart disease category would not enter our burden estimates). Another approach would be to look at other causes [71], or at contributory (i.e., vs. underlying) causes [72]. We do not have an explanation for the sex differences we observe; sex differences in infectious disease in general [73] and influenza in particular [37]–[40] merit further investigation.

One of the biggest challenges of pandemic preparedness is rapid formulation and manufacture of a strain-specific vaccine [74]. Our analysis suggests that younger ages (25–54) should be prioritized in the event of a pandemic. Excess pneumonia and influenza mortality in 2009 peaked at the age group 45–54 for both sexes. The mortality data for the 2009 pandemic do not provide the last word, but do suggest that age-targeted vaccination is a strategy well worth considering.
